# Efficacy of sperm motility after processing and incubation to predict pregnancy after intrauterine insemination in normospermic individuals

**DOI:** 10.1186/1477-7827-11-101

**Published:** 2013-10-22

**Authors:** Lígia FP de Araújo, Edilberto de Araújo Filho, Cássio L Fácio, Márcia CO Bossoni, Ligiane A Machado-Paula, José E Corrente, Mário Cavagna, Paulo CS Matheus, Anaglória Pontes

**Affiliations:** 1 Center of Human Reproduction of São José do Rio Preto, São José do Rio Preto, SP, Brazil; 2 Department of Gynaecology and Obstetrics, Botucatu Medical School, São Paulo State University - UNESP, Botucatu, SP, Brazil; 3 Department of Bioestatistics, Institute of Biosciences Botucatu, São Paulo State University - UNESP, Botucatu, SP, Brazil; 4 Center for Human Reproduction Prof. Franco Jr, Ribeirão Preto, SP, Brazil; 5 Paulista Center for Diagnosis, Research and Training, Ribeirão Preto, SP, Brazil; 6 GERAR - Fertility Clinic, Recife, PE, Brazil

**Keywords:** Sperm motility, Successful insemination, Intrauterine insemination, Sperm survival, Advanced semen analysis

## Abstract

**Background:**

Intrauterine insemination (IUI) is widely used to treat infertility, and its adequate indication is important to obtain good pregnancy rates. To assess which couples could benefit from IUI, this study aimed to evaluate whether sperm motility using a discontinuous gradient of different densities and incubation in CO2 in normospermic individuals is able to predict pregnancy.

**Methods:**

A total of 175 couples underwent 175 IUI cycles. The inclusion criteria for women were as follows: 35 years old or younger (age range: from 27 to 35 years) with normal fallopian tubes; endometriosis grades I-II; unexplained infertility; nonhyperandrogenic ovulatory dysfunction. Men with normal seminal parameters were also included. All patients underwent ovarian stimulation with clomiphene citrate and human hMG or r-FSH. When one or (at most) three follicles measuring 18 to 20 mm were observed, hCG (5000 UI) or r-hCG (250 mcg) was administered and IUI performed 36–40 h after hCG. Sperm processing was performed using a discontinuous concentration gradient. A 20 microliters aliquot was incubated for 24 h at 37 degrees C in 5% CO2 following a total progressive motility analysis. The Mann–Whitney and Chi-square tests, as well as a ROC curve were used to determine the cutoff value for motility.

**Results:**

Of the 175 couples, 52 (in 52 IUI cycles) achieved clinical pregnancies (CP rate per cycle: 29.7%). The analysis of age, duration and causes of infertility did not indicate any statistical significance between pregnancy and no pregnancy groups, similar to the results for total sperm count and morphology analyses, excluding progressive motility (*p* < 0.0001). The comparison of progressive motility after processing and 24 h after incubation between these two groups indicated that progressive motility 24 h after incubation was higher in the pregnancy group. The analysis of the progressive motility of the pregnancy group after processing and 24 h after incubation has not shown any motility difference at 24 h after incubation; additionally, in couples who did not obtain pregnancy, there was a statistically significant decrease in progressive motility 24 h after incubation (*p* < 0.0001). The ROC curve analysis generated a cutoff value of 56.5% for progressive motility at 24 h after incubation and this cutoff value produced 96.1% sensitivity, 92.7% specificity, 84.7% positive predictive value and 98.3% negative predictive value.

**Conclusions:**

We concluded that the sperm motility of normospermic individuals 24 h after incubation at 37 degrees C in 5% CO2, with a cutoff value of 56.5%, is predictive of IUI success.

## Background

Intrauterine insemination (IUI) is considered a non-invasive, less expensive and more acceptable treatment compared with other complex assisted reproduction techniques (ART) such as *in vitro* fertilization (IVF) [1].

There are a variety of indications for using IUI such as unexplained infertility, congenital anomalies of the genital tract, erectile dysfunction, retrograde ejaculation, antisperm antibodies, female sexual dysfunction (vaginismus), cervical factors and infertility caused by endometriosis [2].

The success rate of IUI depends on several factors, including the cause and duration of the infertility, a woman’s age, whether there is controlled ovarian hyperstimulation (COH), the type of medication used and the number of IUI attempts performed previously [3].

Because IUI is a widely used treatment, its adequate indication is crucial to obtaining good pregnancy rates. To obtain this success, severe male factors, tubal and peritoneal factors and severe endometriosis must be excluded [4].

Other important factors that have a strong influence on the IUI outcome include semen quality and several seminal parameters, such as the postwash total motile sperm count (TMC), have been well evaluated as predictors of IUI success [5]. However, studies have shown that the predictive sperm parameters and threshold values that are related to semen characteristics for IUI success are controversial [3,6-18]. Because there is no consensus regarding the ideal limits of these parameters, including sperm concentration and TMC, it is difficult to assess which couples could benefit from IUI. Therefore, most clinicians that specialize in human reproduction often indicate three to six, or more, cycles of IUI before more complex ARTs are attempted [3].

However, among the biological factors assessed, sperm motility is one of the most important parameters in evaluating the fertilizing ability of ejaculated sperm [19]. Two important physiological properties of sperm that are necessary for successful insemination are the number of highly motile sperm and their survival [20]. Studies have demonstrated that motility is considered an important success factor for natural pregnancy as well as for IUI [9,19,21,22].

Knowing the importance of motility, the postwash TMC was proposed as a test for determining which couples could benefit from IUI [6,8]. In addition, the meta-analysis that was published by van Weert *et al.*[5] indicated that the postwash TMC could be used to distinguish patients for either IUI or IVF.

In addition, Branigan *et al.*[20] developed a screening test called “advanced semen analysis”. Using this test, Branigan *et al*. determined the processed motile sperm fraction and assessed sperm survival after 24 h incubation. The results confirmed that this test of sperm motility and survival is predictive of IUI success in oligospermic individuals.

We designed this study because of the need to obtain more answers about which couples could benefit from IUI and because the literature is limited to provide the answers for this important issue. Using the technique developed by Branigan *et al.*[20], we assessed whether sperm motility at 24 h after processing by Isolate discontinuous concentration gradient and incubation at 37°C in 5% CO_2_ in normospermic individuals is able to predict pregnancy.

## Methods

### Study participants

In total, 175 couples underwent 175 IUI cycles from January 2009 to March 2011 in the Center of Human Reproduction of São José do Rio Preto, SP, Brazil, through a longitudinal prospective study.

Selection and inclusion criteria were women 35 years old or younger (age range: from 27 to 35 years), with normal fallopian tubes as determined by hysterosalpingogram and/or videolaparoscopy; endometriosis grades I-II (according to the American Fertility Society); unexplained infertility [23]; nonhyperandrogenic ovulatory dysfunction and men with normal seminal parameters according to WHO [24].

Exclusion criteria included woman with tubal and peritoneal factors; endometriosis grade III-IV [25]; follicle number after ovarian stimulation greater than or equal to four (mean follicular diameter between 18 and 20 mm) on the day of human chorionic gonadotropin (hCG) or recombinant hCG (r-hCG) administration; thickness of endometrium less than 7 mm; unruptured follicle; and cycle cancelation due to non-response to ovarian stimulation.

The local ethics committee (São José do Rio Preto Medical School (FAMERP) - São José do Rio Preto, SP, Brazil) gave institutional review board approval and informed consent was obtained from all couples.

### Methodology

All patients were nuligests and underwent a first cycle of ovarian stimulation with orally administered 50 mg/day clomiphene citrate, from days 3 to 7 of the menstrual cycle, along with subcutaneous human menopausal gonadotropin (hMG, 75 IU) (Menopur; Ferring Ltda, Brazil) or recombinant follicle stimulating hormone (r-FSH, 75 IU) (Gonal F, Serono, Brazil) at days 3, 5 and 7 of the cycle. The follicular development was monitored by transvaginal ultrasound using a 6.5 MHz convex transducer (Midray-Expert 3C5A; China) at days 2 (basal) and 8 of the cycle. Subsequently, daily monitoring was performed until follicular diameter measured 18 to 20 mm and thickness of the endometrium was greater than 7 mm.

When one or (at most) three follicles measured the expected mean diameter, 5000 IU hCG (hCG; Ovidrel, Serono, Brazil) or 250 mcg r-hCG (r-hCG; Ovidrel, Serono, Brazil) was administered. IUI was scheduled 36 to 40 hours after hCG or r-hCG administration and confirmation of follicular rupture.

Semen samples were collected by masturbation after 2–4 days of sexual abstinence. The manipulation of this material was carried out inside the positive pressure laminar flow. Seminal parameters were analyzed by only one researcher and classified according to 2010 WHO criteria [24]. These values for seminal parameters are as follows: volume (ml) ≥ 1.5; sperm concentration (M/ml) ≥ 15; total sperm number (M) ≥ 39 (per ejaculate); total motility (progressive + nonprogressive) (%) ≥ 40; progressive motility (%) ≥ 32; vitality (%) ≥ 58 and morphology (%) ≥ 4 [24].

After semen liquefaction, 10 μl of this sample was placed on a prewarmed (37°C) Makler counting chamber (Sefi-Medical Instruments Ltd) to perform the following analyses using a phase microscope (Eclipse E200 LED - Nikon): sperm concentration/ml, total number of motile and progressive spermatozoa (progressive motility, non-progressive motility and immotility) and morphology. For the outcome analysis only spermatozoa that were classified as progressive motility were considered motile.

After seminal analysis, the sperm processing technique using the Isolate discontinuous concentration gradient (Irvine Scientific, Santa Ana, CA, USA) at room temperature was performed. First 1 ml of 90% density lower layer was added to a 15 ml conical Falcon tube, followed by 1 ml of 50% density upper layer and 1 ml of semen. The sample was then centrifuged at 270 × *g* for 15 minutes.

After centrifugation, the supernatant was removed and the spermatozoa (pellet) was placed into another 15 ml Falcon tube, that contained 5 ml of modified human tubal fluid (mHTF) medium with HEPES (Irvine Scientific), which was supplemented with 15% synthetic serum substitute (SSS, Irvine Scientific), and centrifuged at 270 × *g* for 15 minutes. This process was performed twice.

The final pellet was resuspended in the same medium solution, obtaining a final volume of 1 ml. A 10 μl aliquot was used to perform the post-processing seminal parameter analysis, and a separate 20 μl aliquot was placed into a microtube (Eppendorf) and incubated (Forma Scientific, INC. Model 3110) for 24 h at 37°C in 5% CO_2_. Only the sperm concentration of the 20 μl aliquot was adjusted to a total 10×10^6^ motile sperm per milliliter of medium. The 970 μl of remaining semen was used to perform IUI using an intrauterine Frydman 5.5 catheter (Frydman Classic Catheter 1306055, CCD Laboratorie Paris-France), by the same physician. The patient was on bed rest for 20 minutes, and the supplementation of the luteal phase was administered orally with natural progesterone at a dose of 200 mg every 12 h from the day after IUI until confirmation of the embryonic heartbeat using an ultrasound.

After 24 h incubation, a progressive motility analysis was performed. IUI success (clinical pregnancy - CP) was confirmed by the presence of a gestational sac with an embryonic heartbeat at 14 days after positive beta-hCG.

Couples were divided in two groups (pregnancy and no pregnancy) according to results of the IUI.

### Statistical analysis

The sample size calculation was performed using the method previously described by Branigan *et al.*[20], i.e., this calculation was carried out supposing a proportion of 70% of fertilization after incubation for 24 hours, with a 95% confidence coefficient and a 7% error margin with a minimum of 164 couples. The results were presented as medians, quartiles and percentages. The data distribution of this study was asymmetric. The Mann–Whitney test was used to compare women’s ages and infertility parameters, as well as the initial analysis of the concentration, morphology and motility between pregnant and nonpregnant individuals. The chi-square test (χ^2^) for differences in proportions was used to compare the percentage of motility for pregnant and nonpregnant individuals.

A receiver operating characteristic curve (ROC) was constructed to determine which cutoff value for post-processing spermatic motility and incubation for 24 h in CO_2_ predicts pregnancy after IUI in normospermic individuals. The sensitivity, specificity, positive predictive value (ppv) and negative predictive value (npv) were calculated for two variables: after processing and incubation for 24 h with their respective 95% confidence intervals (CIs). Sensitivity was defined by the presence of pregnancy when it actually occurred, and the specificity was defined as the absence of pregnancy when it did not occur. The ppv is the probability of pregnancy with motility after incubation with the determined cutoff value and npv is no pregnancy probability after incubation with this same cutoff value.

All statistical analyses were performed using the SAS 9.2 for Windows and SPSS for Windows V.19 software. The level of 5% or the corresponding *p*-value was considered for statistically significant in all tests.

## Results

Of the 175 couples, 52 (in 52 IUI cycles) achieved CP (CP rate per cycle: 29.7%) and 123 did not have success (70.3%). The rate of hospital-discharged live births was 27.4% (48 of 175). There were 4 spontaneous abortions (7.6%) (three cases occurred during the first-trimester and one during the 20th week of gestation) and one case of multiple pregnancy (1.9%).

The clinical characteristics of couples who underwent IUI are shown in Table 1. The median age of pregnant and nonpregnant women after IUI was 28 and 31 years respectively. The range of women’s age in the pregnancy group was from 28 to 35 years and in the no pregnancy group was from 27 to 35 years. The comparison of age, duration and causes of infertility did not show any statistical significance between pregnancy and no pregnancy groups, similar to the results for total sperm count and morphology, except progressive motility (*p* < 0.0001).

**Table 1:** Clinical characteristics and seminal parameters of couples who underwent IUI according to pregnancy or no pregnancy

**Variables**	**Pregnancy (n = 52)**	**No pregnancy (n = 123)**	***p-value *** *
Women’s age (years)	28 (28–32)	31 (30–32)	0.065
Men’s age (years)	33 (31–36)	34 (32–37)	0.102
Duration of infertility (years)	2 (1.6-3.5)	3 (2–4)	0.209
**Etiology (%)**			
Unexplained infertility	25.0	29.0	0.56 **
Endometriosis	25.2	23.6	
Cervical factor	23.1	16.3	
Ovulatory dysfunction	30.8	24.4	
Total sperm count (×10^6^)	200 (72–310)	150 (80–300)	0.72
Morphology (%)	31 (25–36)	30 (22–35)	0.37
Progressive motility (%)	52 (38–60)	35 (32–49)	0.0001

Values are expressed as median and quartiles.

*= Mann–Whitney test.

**= χ^2^ analysis to access etiology of infertility.

In Table 2, the comparison of progressive sperm motility after processing on the day of IUI day and at 24 h after incubation between pregnancy and no pregnancy groups is presented. We observed that, in couples who underwent IUI, there was no significant differences in the post-processing sperm motility between both groups (*p* = 0.4807). However progressive motility at 24 h after incubation was increased in the pregnancy group. The difference between median and quartiles values were very apparent. The analysis of progressive sperm motility in the pregnancy group indicated no significant difference after processing (70.5%; 95% CI: 67.5-77%) and after incubation (70%; 95% CI: 67-73%) (*p* = 1.0). In the no pregnancy group, there was a significant decrease in progressive sperm motility at 24 h after incubation, 65% (95% CI: 44-77%) to 24% (95% CI: 12-41%) (*p* < 0.0001).

**Table 2:** **Comparison between progressive motility after sperm processing and incubation for 24 h at 37°C in 5% CO**_**2**_

**Progressive motility (%)**	**Pregnancy (n = 52)**	**No pregnancy (n = 123)**	***p-value *** *
After sperm processing	70.5 (67.5-77)	65 (44–77)	0.4807
After 24 h incubation	70.0 (67–73)	24 (12–41)	<0.0001
***p-value***	1.000	<0.0001	

Values are expressed as percentage and 95% CI.

* = χ^2^ analysis with significant difference at *p* < 0.05.

**Figure 1:**
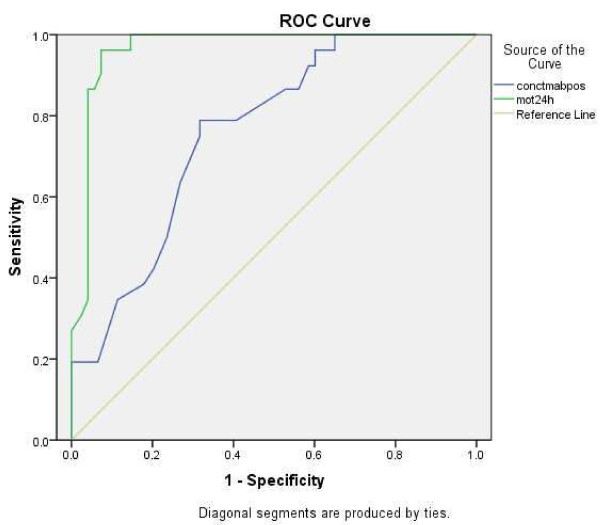
**ROC curve.** ROC curve analysis for motility after sperm processing (area under the curve: 0.759) and at 24 h after incubation at 37°C in 5% CO_2_ (area under the curve: 0.965) as a predictive factor to clinical pregnancy after IUI in normospermic individuals. Blue line: conctmabpos = total concentration of progressive motility after sperm processing. Green line: mot24h = motility at 24 h after incubation.

The ROC curve (Figure 1) determined the cutoff value for sperm motility after processing and at 24 h after incubation as the predictive factor for pregnancy in normospermic individuals. The area under the curve was 0.965 for the 56.5% cutoff value, and 0.759 for the 69% cutoff value.

Therefore, through the ROC curve analysis, a cutoff value was generated for greater sensitivity and specificity for pregnancy. A cutoff value of 56.5% with 96.1% sensitivity (95% CI: 90.9-100%) and 92.7% specificity (95% CI: 88.1-97.3%) was obtained for progressive motility at 24 h after incubation. The sensitivity and specificity, ppv and npv at 24 h after incubation were increased compared with the values for after sperm processing, taking into account the ROC curve cutoff values of 56.5% and 69%, respectively (Table 3).

**Table 3:** Sensitivity, specificity, positive and negative predictive values as determined by cutoff values of the ROC curve

	**After sperm processing (cutoff value: 69%)**	**24 h incubation (cutoff value: 56.5%)**
**Sensitivity**	63.5 (50.4 – 76.5)	96.1 (90.9 – 100.0)
**Specificity**	73.1 (65.0 – 81.0)	92.7 (88.1 – 97.3)
**Positive predictive value**	50 (37.9 – 62.1)	84.7 (75.5 – 93.9)
**Negative predictive value**	82.6 (75.5 – 89.7)	98.3 (95.9 – 100.0)

Values are expressed as percentage and 95% CI.

## Discussion

IUI is a widely used resource for the treatment of infertile couples, with great chances for success when IUI is properly selected. Semen quality is one of the factors that contributes to the technique’s success, and among the semen parameters, sperm motility is considered an important factor for obtaining high pregnancy rates in couples that have indications for IUI treatment [22]. Several studies have shown that progressive motility and/or total motile sperm [5-7,9,10,13,14,16,21,22],[26-28] were considered the best predictors of pregnancy after sperm processing.

Pasqualotto *et al.*[28] assessed 504 couples who underwent IUI to investigate the relation between the postwash TMC and postwash percentage of motility as well as to determine the minimal postwash TMC as a predictive factor of IUI success. The results indicated that, independent of the postwash TMC, the postwash motility predicted IUI success at a cutoff of 40%.

Van Weert *et al.*[5] performed a meta-analysis (16 studies) to investigate the performance and clinical value of the postwash TMC as a test to predict IUI outcome. Their results indicate that the value of the postwash TMC at insemination relies on the enhancement of patient selection by identifying couples that are unlikely to conceive with IUI and not on the selection of patients that are most likely to conceive. This result occurred because there was high specificity (failure to become pregnant) of the postwash TMC, with different cutoff values for different clinics. These cut-off values probably were variable due to the different methodologies of sperm preparation in each study. However, the authors concluded that the postwash TMC could be used in counseling patients for the selection of the better treatment, either IUI or more complex methods.

When we compare our data with the literature, we believe that the success rate that was achieved in the present study (29.7% of CP rate per cycle) may be due to the adequate indication of IUI, exclusion of male and severe female factors, and to the fact that the sperm concentration and the number of motile sperm for IUI was according to the WHO reference values [24].

Demir *et al.*[29] evaluated the effect of different sperm parameters on the pregnancy rate of IUI cycles in women with favorable fertility characteristics that were treated for infertility. Demir *et al.* assessed 212 infertile couples and obtained a pregnancy rate of 15.8% per cycle. The woman’s age and total number of motile sperm were predictive factors for pregnancy. The pregnancy rate was the highest in IUI cycles when woman were <25 years old, the total number of motile sperm was >10 million and morphology was >4%. Male age was also a determining factor for IUI success, even with a normal spermiogram. In our study, there was no significant difference in age (both men and women) in both groups (pregnancy and no pregnancy), and our data reinforce that sperm motility, which was verified in the present study, may help patients determine their chances of IUI success.

Currently, many studies investigate seminal parameters and its importance in achieving good pregnancy outcomes in couples undergoing IUI. However many controversies make it impossible to know which couples could benefit from this form of treatment because the basic semen analysis is not a good parameter for IUI success. Moreover, we do not have answers regarding the feasibility of selectively insisting on the IUI technique. There is only one study in the literature, which was performed by Branigan *et al.*[20], that analyzed sperm motility at 24 h after processing and incubation as the predictive test of IUI success. According to the study outcome, this motility test is predictive of IUI success. The authors assessed 414 couples undergoing IUI because of a male factor and unexplained infertility. Their results showed that none of the basic semen analysis parameters of concentration, motility or morphology was predictive of IUI success. With the advanced analysis, when the processed total motile sperm that were available for IUI was ≥10 × 10^6^ and the sperm survival at 24 h after incubation was ≥70%, 89% of the couples achieved pregnancy, with a 21.4% pregnancy rate per cycle. With the cutoff values >70% for the advanced semen analysis, the test had a sensitivity of 94% and specificity of 86%.

Interestingly the authors compared men with normal semen analysis (group of unexplained infertility) with those men presenting a male factor. The results showed that 83% of couples had male factor problems (basic semen analysis parameters), presenting a per cycle pregnancy rate of 17.8% and a 48% cumulative pregnancy rate. Twenty-three percent of men with normal semen parameters presented a per cycle pregnancy rate of 1.8% and they did not meet the advanced semen analysis cutoff values for 24 h motility or processed total motile sperm. Therefore, the advanced test was predictive, independent of normal or abnormal the basic analysis seminal parameters, because this advanced analysis accurately predicted these “occult” male factors.

This same type of test was performed in this work, but only in normospermic individuals, which allowed a good success rate in our IUI results. Some studies confirmed that pregnancy rates are higher in normospermic individuals as shown by Allen *et al.*[30], which indicated a 25% pregnancy rate per cycle in 104 couples when the male factor was predominant and a 60% pregnancy rate per cycle in 58 couples when there was a cervical factor. For Grigoriou *et al.*[31], the rates of CP and live births per cycle were significantly lower in the teratozoospermia group when compared with the normozoospermia group, whereas the cumulative live birth rate after 4 IUI cycles was significantly lower in the group with male factors of infertility.

These data corroborate the results presented here, indicating that high pregnancy rates are obtained in normospermic patients in which sperm motility after incubation remains unchanged. Therefore, the test with 24 h incubation at 37°C in 5% CO_2_ after sperm processing allowed us to conclude that motility is related to the success rate of IUI. Thus, couples seeking treatment for infertility could be subjected to this test, and from the test results, we would be able to select those couples who might benefit from IUI. Thus, couples avoid subjection to several unnecessary cycles of IUI. Successive failed attempts of the IUI can be a frustrating experience for the couple, not only by the distress between one cycle and another but also by the fear of not achieving success [10].

We also believe that if this predictive test of IUI success was applied to patients before treatment, then a consensus could be determined regarding the existing variation in the limit for the number of cycles to be performed. The present study aims at this goal through a cutoff value using motility, which is the parameter that appears to be the most relevant. This observation is consistent with the studies by Shulman *et al.*[22], who concluded that the degree of sperm motility is the only parameter that can be correlated with the IUI outcome for normal women with a partner with good sperm motility, after appropriate sperm preparation.

The IUI is a less complex treatment and the test of sperm motility at 24 h after incubation at 37°C in 5% CO_2_ can be used to predict CP prior to the IUI treatment or as an indicator to verify whether a new IUI attempt must be performed or more complex ARTs are required.

## Conclusions

In conclusion, the present study suggests that determining the sperm motility of normospermic individuals after processing and incubation for 24 h at 37°C in 5% CO_2_, with a cutoff value of 56.5% is able to predict IUI success.

## Abbreviations

ART: Assisted reproduction techniques; CI: Confidence interval; COH: Controlled ovarian hyperstimulation; CP: Clinical pregnancy; hCG: Human chorionic gonadotropin; hMG: Human menopausal gonadotropin; IUI: Intrauterine insemination; IVF: *in vitro* fertilization; mHTF: Modified human tubal fluid; npv: Negative predictive value; ppv: Positive predictive value; r-FSH: Recombinant follicle stimulating hormone; r-hCG: Recombinant human chorionic gonadotropin; ROC: Receiver operating characteristic curve; SSS: Synthetic serum substitute; TMC: Total motile sperm count.

## Competing interests

The authors declare that they have no competing interests.

## Authors’ contributions

LFPA was responsible for designing and coordinating the study, the collection, analysis and interpretation of the data, and for writing the manuscript. EAF was responsible for the collection, analysis and interpretation of the data. CLF and MCOB were responsible for data collection. LAM-P was responsible for the analysis and interpretation of the data and revising the manuscript. JEC was responsible for the statistical analyses. MC and PCSM were responsible for revising the manuscript. AP was responsible for designing and coordinating the study. All authors read and approved the final manuscript.
